# pS421 huntingtin modulates mitochondrial phenotypes and confers neuroprotection in an HD hiPSC model

**DOI:** 10.1038/s41419-020-02983-z

**Published:** 2020-09-25

**Authors:** Xiaohong Xu, Bryan Ng, Bernice Sim, Carola I. Radulescu, Nur Amirah Binte Mohammad Yusof, Wah Ing Goh, Shuping Lin, John Soon Yew Lim, Yoonjeong Cha, Rebecca Kusko, Chris Kay, Tamara Ratovitski, Christopher Ross, Michael R. Hayden, Graham Wright, Mahmoud A. Pouladi

**Affiliations:** 1 Department of Neurology and Stroke Center, Clinical Neuroscience Institute, The First Affiliated Hospital, Jinan University, 613 Huangpu Avenue West, Guangzhou Guangdong, 510632 China; 2 Translational Laboratory in Genetic Medicine, 8A Biomedical Grove, Immunos, Level 5, Singapore, 138648 Singapore; 3 Institute of Medical Biology, 8A Biomedical Grove, Immunos, Level 5, Singapore, 138648 Singapore; 4 Immuneering Corporation, 157 Columbus Avenue, Suite 537, New York, NY 10023 USA; 5 Centre for Molecular Medicine and Therapeutics, University of British Columbia, Vancouver, V5Z4H4 Canada; 6 Division of Neurobiology, Department of Psychiatry and Behavioral Sciences, Johns Hopkins University School of Medicine, Baltimore, MD 21205 USA; 7 Department of Medicine, National University of Singapore, Singapore, 117597 Singapore; 8 Department of Physiology, National University of Singapore, Singapore, 117597 Singapore

**Keywords:** Cell death in the nervous system, Induced pluripotent stem cells

## Abstract

Huntington disease (HD) is a hereditary neurodegenerative disorder caused by mutant huntingtin (mHTT). Phosphorylation at serine-421 (pS421) of mHTT has been shown to be neuroprotective in cellular and rodent models. However, the genetic context of these models differs from that of HD patients. Here we employed human pluripotent stem cells (hiPSCs), which express endogenous full-length mHTT. Using genome editing, we generated isogenic hiPSC lines in which the S421 site in mHTT has been mutated into a phospho-mimetic aspartic acid (S421D) or phospho-resistant alanine (S421A). We observed that S421D, rather than S421A, confers neuroprotection in hiPSC-derived neural cells. Although we observed no effect of S421D on mHTT clearance or axonal transport, two aspects previously reported to be impacted by phosphorylation of mHTT at S421, our analysis revealed modulation of several aspects of mitochondrial form and function. These include mitochondrial surface area, volume, and counts, as well as improved mitochondrial membrane potential and oxidative phosphorylation. Our study validates the protective role of pS421 on mHTT and highlights a facet of the relationship between mHTT and mitochondrial changes in the context of human physiology with potential relevance to the pathogenesis of HD.

## Introduction

Huntington disease (HD), a devastating hereditary neurodegenerative disorder, is caused by an expansion of a CAG trinucleotide repeat that encodes a polyglutamine (polyQ) tract in the huntingtin (*HTT*) gene. HTT is a ubiquitously expressed 350 kDa protein that is found in different subcellular compartments, including the nucleus, mitochondria, the Golgi complex, microtubules, and vesicular structures in neurites and synapses. Proposed to act as a scaffold^1^, HTT interacts with a large number of proteins. Posttranslational modifications (PTMs) of HTT are thought to be key determinants of HTT’s conformation as well as its ability to associate with its interacting partners. These PTMs have been shown to include phosphorylation, acetylation, SUMOlyation, proteolytic cleavage, palmitoylation, and ubiquitination^2,3^. The toxicity of the expanded polyQ tract is postulated to result, at least in part, from disrupted interactions with molecular partners as a consequence of altered PTMs of HTT.

Converging lines of evidence support a role for phospho-S421 (pS421) levels in modulating mutant HTT (mHTT) toxicity. The pro-survival signaling protein Akt is one of the kinases shown to phosphorylate HTT on S421^1,4^. pS421-HTT levels show a regional distribution that is inversely correlated with neuronal atrophy in HD, with the highest levels in the cerebellum, less in the cortex, and least in the striatum^5^. pS421-HTT levels decline steadily over time in the striatum of YAC128 HD mice^6^. A number of cellular defects have been linked to the reduced levels of pS421-HTT, including intracellular transport defects^7^, increased proteolysis and nuclear accumulation of HTT fragments^8^, and diminished neuronal survival^6,9^.

Although previous studies have explored the impact of PTMs on mHTT function and toxicity, the interpretation of the findings from these studies is confounded by caveats that include the following: (a) the use of truncated N-terminal fragments of mHTT that comprise only 67 to ~700 amino acids (aa) of the 3144 aa full-length protein; (b) the use of non-endogenous promoters typically resulting in expression levels that are several fold higher than the endogenous mHTT levels; and (c) the use of non-neuronal cell lines such as HEK293 or HeLa cells, or rodent-derived neuronal cell lines such as mouse Neuro2a cells^10–13^. Therefore, understanding the importance of PTMs in the context of human pathology remains a major gap in our knowledge of the significance of HTT PTMs specifically and the pathogenesis of HD in general.

Here we sought to examine the impact of pS421 of HTT on expanded polyQ-related phenotypes in human induced pluripotent stem cell (hiPSC)-derived neural cells. Using a TALEN (transcription activator-like effector nuclease)-based genomic editing approach, we mutated polyQ-expanded HTT at S421 to create a variant that is resistant to phosphorylation at S421 (a serine to alanine substitution, S421A) or one that mimics phosphorylation (a serine to aspartic acid substitution, S421D). The functional consequences of the phosphorylation-competent polyQ-expanded HTT (S421S) were compared to phosphorylation-resistant polyQ-expanded HTT (S421A) and pseudo-phosphorylated polyQ-expanded HTT (S421D) in hiPSC-derived neural cells. We observed that S421D, rather than S421A, confers neuroprotection against mHTT toxicity in hiPSC-derived neural cells. We further found that this neuroprotection was associated with modulation of several aspects of mitochondrial form and function including mitochondrial surface area, volume, and counts, as well as improved mitochondrial membrane potential and oxidative phosphorylation. Our study highlights a facet of the relationship between mHTT and mitochondrial changes in the context of human physiology with potential relevance to the pathogenesis of HD. Furthermore, the approach we describe here can be applied widely to investigate the effect of other PTMs on neurodegenerative disease phenotypes in a genetically faithful and physiologically relevant human context.

## Materials and methods

### Cloning of constructs

S421-T1 and S421-T2 TALENs were cloned into pCDNA4 vector by a single step of the 4 × 4 Golden Gate assembly protocol using a premade tetramer TALE repeat library by the Biological Resource Centre, A*STAR, Singapore. Subsequently, S421-T1 and S421-T2 TALENs were cloned into pTAL-GFP (Addgene, #43858) and pTAL-RFP (Addgene, #43859) vector using BamHI and Xbal restriction enzymes, respectively. Single-stranded oligodeoxynucleotides (ssODNs) donors for S421 site targeting were synthesized by Sigma-Aldrich. S421-T1 and S421-T2 TALEN-binding sequence and ssODN sequence are listed in Supplementary Table S1.

### Cell culture

HEK293 cells for TALENs activity test were cultured in Dulbecco’s modified Eagle medium (DMEM) supplemented with 10% fetal bovine serum (FBS). K562 cells for ssODNs donor test were cultured in Iscove’s modified Dulbecco’s medium supplemented with 10% FBS. CAG180 hiPSCs (ND36999) and CAG33 hiPSCs (ND36997) were obtained from the NINDS hiPSC Repository at Coriell Institute. HD-C hiPSCs are isogenic lines established by correction of mutant CAG180 repeats with 18 CAG repeats as described previously^14^. All hiPSCs were cultured on Matrigel (BD Biosciences)-coated plates with mTesR-1 medium (Stemcell Technologies, #05850).

### Differentiation of hiPSCs

#### NPC differentiation

hiPSCs were induced into neural progenitor cells (NPCs) according to a previously published protocol^15^. Briefly, hiPSCs at ~20% confluence were treated with N2B27 media (DMEM-F12/Neural Basal medium 1 : 1 with 1% N2, 2% B27, 1% pen/strep/glutamine, 10 ng/mL hLIF, and 5 μg/mL bovine serum albumin) containing 3 μM CHIR99021 (Tocris), 2 μM SB431542 (Sigma), and 0.1 μM compound E (Millipore) for the first 7 days. The culture was then split 1 : 3 for the next six passages using Accutase without compound E on Matrigel-coated plates. Cells on passage 3 and passage 4 were used for experiments.

#### Forebrain neuronal differentiation

hiPSCs were differentiated into forebrain neurons using our previously published protocol^14,16^. Briefly, NPCs were induced in N2B27 medium supplemented with certain small molecules and growth factors (GFs) for 15 days. For final neuronal differentiation, the cells were cultured in N2B27 medium supplemented with brain-derived neurotrophic factor (BDNF) (20 ng/ml), glial cell line-derived neurotrophic factor (GDNF) (20 ng/ml), dbcAMP (0.5 mM), and Ascorbic Acid (0.2 mM). Half the medium was replaced with fresh medium every 3–4 days for the terminally differentiated neurons.

#### Cortical neuron differentiation

Differentiation of hiPSCs into cortical neurons was performed as previously described^17^. The cortical neurons on Day 53 were dissociated and re-plated on glass-bottom dishes for mitochondrial axonal transport analysis.

### YOYO-1 cell death assay

NPCs were dissociated into single cells using Accutase and seeded onto Matrigel-coated 96-well plate and cultured overnight. Next day, cells were first treated with 200 µM tert-butyl hydroperoxide (TBHP) for 2 h and switched to fresh NPC medium containing 100 nM YOYO-1 (Thermo Fisher Scientific, catalog number Y3601). Imaging was performed for 24 h using the Incucyte Zoom system (Essen Bioscience). YOYO-1 expression levels on 24 h were normalized to the 0 h expression levels for each well and the data from two individual clones per genotype were combined together for statistical analysis.

### MitoTracker staining and flow analysis

NPCs were stained with 100 nM MitoTracker Green FM (Invitrogen, M7514) and 100 nM MitoTracker Red CMXRos (Invitrogen, M7512) for 15 min at 37 °C, according to the manufacturer’s instructions. The stained cells were run on CytoFLEX flow cytometer (Beckman Coulter) and the mean fluorescence intensity were used for analysis.

### Mitochondrial respiration analysis

The NPCs were plated on the 96-well assay plate at a density of 150,000 cells/well 1 day before assay. To measure the mitochondrial respiration under oxidative stress conditions, cells were treated with 200 µM TBHP for 2 h and switched to DMEM/F12 medium for another 2 h before assay. The assay was carried out using a Seahorse XF96 Extracellular Flux Analyzer followed the instructions of Seahorse XF Cell Mito Stress Test Kit (Seahorse Bioscience). The readings were normalized to total protein amount measured by Bradford assay and data were analyzed using the Seahorse Wave software.

### RNA-Seq analysis

RNA was extracted from cells using a RNeasy plus mini kit (Qiagen), according to the manufacturer’s instructions. Subsequent library preparation and paired-end 100 bp sequencing and 30 M reads/per sample using Illumina HiSeq4000-PE150 were performed by Novogene (Hong Kong). Gene set enrichment analysis (GSEA) was used to test whether the gene expression signatures of pS421-mHTT were enriched for Kyoto Encyclopedia of Genes and Genomes, Reactome, and Gene Ontology Biological Process pathways^18–20^. The RNA sequencing (RNA-Seq) data were deposited into Sequence Read Archive (SRA) with the Bioproject ID of PRJNA474894. See Supplementary Information for the details of the protocol.

### Measurement of mitochondrial morphology

Cells were fixed and immunostained with anti-TOM20 (1 : 500; Proteintech, 11802–1-AP) primary antibodies and Alexa Fluor 568-conjugated secondary antibodies to visualize the mitochondrial network. A DeltaVision OMX v4 Blaze microscope (GE Healthcare, Issaquah, WA, USA) was used for acquisition of widefield-deconvolved images of individual NPCs. Images were acquired at a *z*-spacing of 0.125 µm as previously described^21^. To measure mitochondrial morphological parameters, the deconvolved widefield images were imported into IMARIS (Bitplane) for three-dimensional (3D) reconstruction. Raw data points of each mitochondrion detected were exported for statistical analysis. See Supplementary Information for the details of the protocol.

The detailed methods for nucleofection of hiPSCs, screening targeted clones, allele-specific PCR, fragment sizing analysis, RNA isolation, cDNA synthesis and quantitative PCR, CellTiter-Glo® luminescent cell viability assay, GF withdrawal assay, measurement of mitochondrial morphology, immunoblotting, immunofluorescence staining, and RNA-Seq analysis are found in the Supplementary Materials and Methods.

### Statistics

For experiments with hiPSC-derived neural cells, each biological replicate was derived from an independently differentiated clone and subsequent assessment. All statistical analyses were performed in GraphPad Prism 7 software. One-way analysis of variance was used to test for differences across different groups, followed by a Fisher’s least significant difference post hoc multiple-comparison test to test for specific differences between groups. Otherwise, for comparisons of the mean between two groups, Student’s *t*-test was applied. All the data were shown as mean ± SEM and *p*-values were considered as follows: ns, no significance; *p < 0.05, **p < 0.01, and ***p < 0.001.

## Results

### Generation of isogenic S421A/D-mHTT hiPSCs

**Fig. 1:**
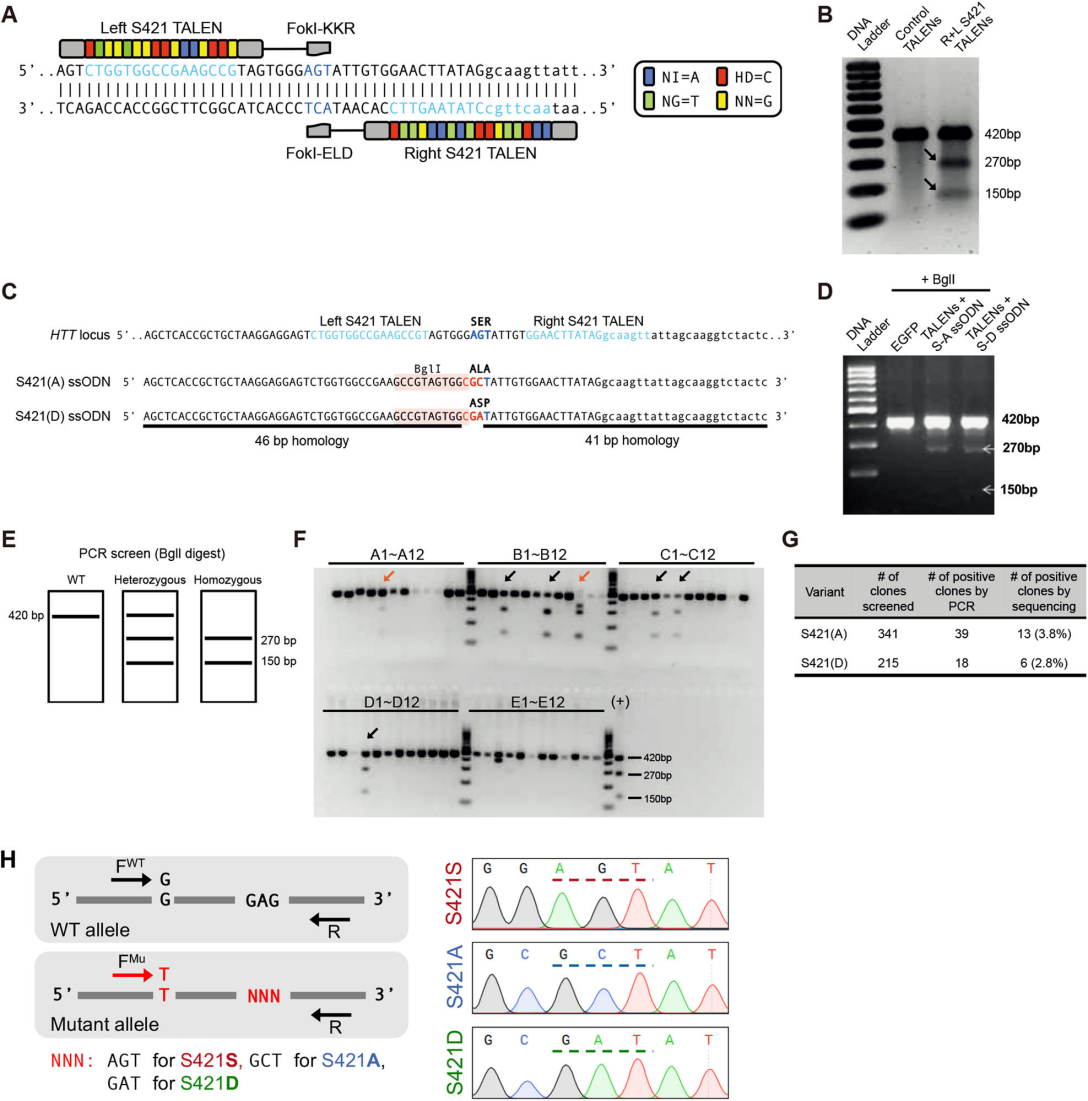
Genome-editing strategy to generate isogenic S421A/D-mHTT hiPSC lines. **a** TALEN-binding sequences flanking the S421 codon in HTT exon 9. **b** TALEN cleavage activity as assessed by Surveyor assay. **c** ssODN donor DNA with BglI restriction enzyme site for screening. **d** Verification of targeting and screening strategy in K562 cells. **e** Expected restriction fragment pattern with BglI screening. **f** HTT Exon 9 PCR followed by BglI screening identifies a number of targeted CAG180 HD hiPSC clones (black arrows); example shown is for S421(A) targeting; red arrows represent mixed clones. **g** Summary of targeting efficiency. **h** Phasing of targeted allele by allele-specific PCR followed by Sanger sequencing.

A published CAG180 HD hiPSC line (Coriell, ND36999), demonstrating certain distinct HD phenotypes^14,22^, was chosen as the parental cell line to generate isogenic S421A and S421D HD hiPSC lines. We designed a pair of TALENs, which could bind to the DNA sequences around the S421 site (Fig. 1a; S421-T1/2) with very few off-target sites predicted by TAL Effector Nucleotide Targeter 2.0 (Table S2). S421-T1 and S421-T2 TALENs demonstrated efficient cleavage activity assessed by surveyor assay in HEK293 cells (Fig. 1b). To mutate the serine at S421 site into phospho-mimetic aspartic acid (S421D) or phospho-resistant alanine (S421A), 90 bp ssODNs were used as donors. The ssODNs contained two nucleotides “GC/GA” mutations for converting “S” into “A” or “D” at the S421 site, respectively, and one additional synonymous “C” nucleotide mutation to introduce a BglI restriction enzyme site to facilitate targeted clone screening (Fig. 1c). The homology-directed repair by ssODNs and screening strategy were validated in human K562 cells (Fig. 1d). As illustrated, the PCR products after BglI digestion were predicted to result in one band for non-targeted clones, two lower bands for targeted homozygous clones, and three bands for heterozygous or mixed clones (Fig. 1e). Using this strategy, we electroporated green fluorescent protein (GFP)-tagged S421-T1, red fluorescent protein (RFP)-tagged S421-T2 TALENs, and ssODNs into CAG180 hiPSCs. The GFP(+)/RFP(+) hiPSCs were sorted into 96-well plates for clone expansion and PCR screening (Fig. 1f). The targeting efficiency was around 3% in CAG180 hiPSCs by PCR screening (Fig. 1g). To determine the targeted allele, we employed allele-specific PCR using a known single-nucleotide polymorphism upstream of S421 site on the mutant allele in CAG180 line (Fig. 1h). Moreover, the clones that had undergone the whole targeting process without modification on S421 sites were used as control clones (referred to as S421S) and clones only targeted on the mutant allele were named as S421A or S421D. All candidate subclones of S421S, S421A, and S421D iPSCs for further validation were heterozygous and at least two subclones per genotype groups were used for subsequent experiments.

We then examined the pluripotency of the targeted clones by OCT4 staining and mRNA expression of pluripotent marker OCT4 and LIN28. All lines had similar expression levels of OCT4 and LIN28 compared with the parental CAG180 HD hiPSC line (Supplementary Fig. S1a, b). Karyotyping and G-banding analysis demonstrated that all clones maintained a normal 46, XY karyotype (Supplementary Fig. S1c) and they demonstrated similar CAG expansion size and pattern via CAG fragment sizing analysis (Supplementary Fig. S1d).

### S421 phosphorylation status influences susceptibility to cell death

**Fig. 2:**
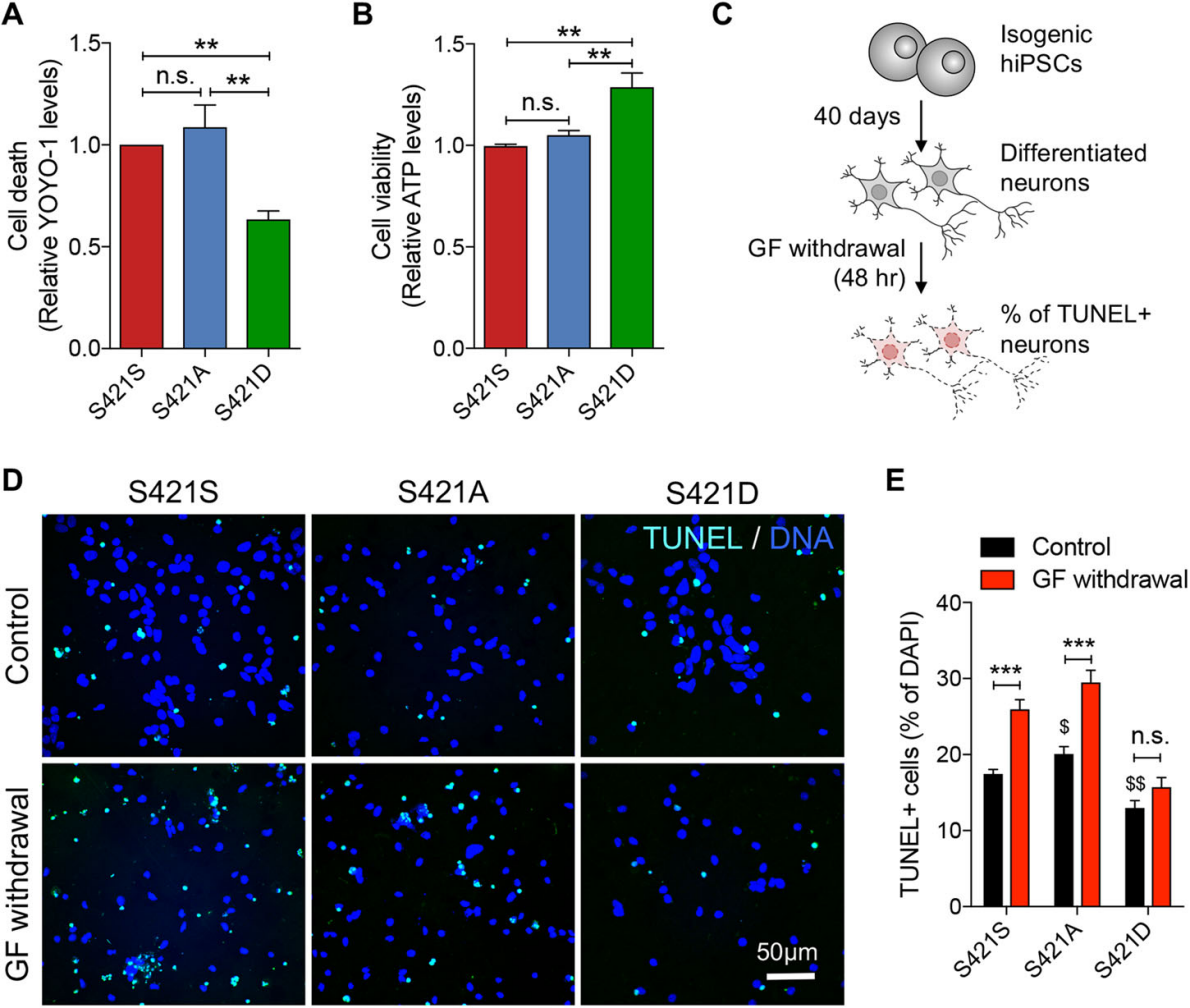
Phosphorylation status of S421-mHTT influences susceptibility to cell death. **a** TBHP-induced cell death in neural progenitor cells (NPCs) measured using the YOYO-1 cell death assay. *n* = 3 independent biological replicates (BRs). **b** Cell viability of neural progenitor cells following TBHP treatment determined using the ATP assay. *n* = 3 BRs. **a**, **b** n.s., no significance and **p < 0.01 was determined by one-way ANOVA analysis followed by a Fisher’s LSD post hoc multiple-comparison test. **c** Schematic of neuronal death assay after growth factor withdrawal. **d** TUNEL immunofluorescence images showing cell death in S421S, S421A, and S421D neurons following growth factor withdrawal. **e** Quantification of cell death as measured by TUNEL staining in S421S, S421A, and S421D neurons following growth factor withdrawal. *n* = 6 for 2 clones per genotype with 3 BRs. Two subclones were used per genotype group. Values shown as mean ± SEM and n.s., no significance, *p < 0.05 and ***p < 0.001 for shown comparisons, and ^$^*p* < 0.05 and ^$$^*p* < 0.01 relative to S421S-Control was determined by unpaired *t*-test.

To validate the neuroprotective role of S421D in iPSC-derived neural cells, we first examined the susceptibility of NPCs derived from S421S, S421A, and S421D HD hiPSCs to oxidative stress. NPCs were treated with 200 µM TBHP for 2 h, after which cells were cultured for another 24 h before being assayed. YOYO-1, a green fluorescence dye that stains the nuclear DNA of cells that have lost plasma membrane integrity^23^, was used to quantify cell death. S421D NPCs showed less cell death after TBHP treatment compared to S421S and S421A NPCs (Fig. 2a). Consistently, cell viability of S421D NPCs after TBHP treatment as measured by CellTiter-Glo assay was improved as compared to that of the S421S and S421A lines (Fig. 2b).

We next sought to determine whether the S421 phosphorylation status would influence the susceptibility of post-mitotic HD neurons to GF withdrawal, as we previously observed that HD neurons were more susceptible to cell death after GF withdrawal compared with corrected isogenic control neurons^14^. Post-mitotic neurons differentiated from hiPSCs on Day 40 were used for the GF withdrawal assay (Fig. 2c and Supplementary Fig. S2). Under normal culture conditions with GF, the S421A showed an increased cell death, whereas S421D neurons had less cell death compared with S421S neurons (Fig. 2d, e). Moreover, GF withdrawal resulted in increased cell death of S421S and S421A neurons, in contrast to S421D neurons, which did not show any increase in cell death following GF withdrawal (Fig. 2d, e).

In summary, we observed that S421D, the pseudo-phosphorylated status, rather than phospho-resistant S421A, protected HD hiPSC-derived NPCs and neurons against cell death caused by oxidative stress and GF withdrawal, respectively.

### S421 phosphorylation status does not affect proteasome-dependent clearance of mHTT, axonal transport, or neural rosette formation

**Fig. 3:**
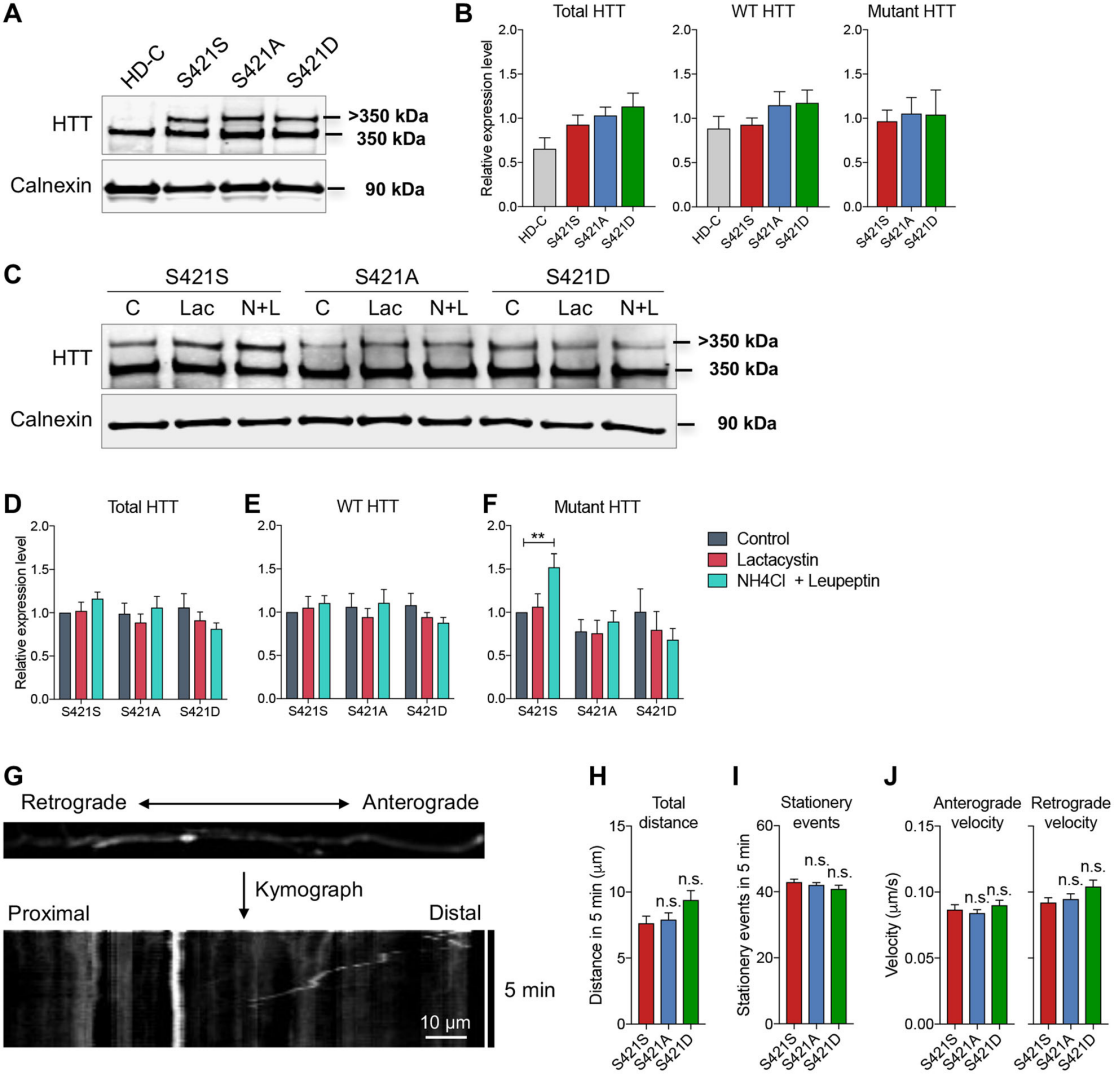
No effect of S421A/D on proteasome-dependent clearance of HTT or axonal transport. **a**, **b** Expression levels of HTT in hiPSCs by immunoblotting (**a**) and quantification of total HTT, WT HTT, and mutant HTT (**b**). HD-C is a corrected isogenic line for parental CAG180 HD iPSC line. **c**–**f** HTT clearance assay in NPCs derived from isogenic S421S, S421A, and S421D HD hiPSCs. C, Control; Lac, 1 μM Lactacystin; N + L, 200 nM NH4Cl and 10 μM Leupeptin. Representative immunoblotting image (**c**) and quantification of total HTT (**d**), WT HTT (**e**), and mutant HTT (**f**). Values shown as mean ± SEM and *p < 0.05 for shown comparisons was determined by one-way ANOVA followed by a Fisher’s LSD post hoc multiple-comparison test. *n* = 6 for 2 clones per group from 3 BRs. **g** Schematic of mitochondrial transport analysis. **h** Total vesicle moving distance in 5 min, *n* = 76 for S421S, 70 for S421A, and 77 for S421D. **i** Stationary events per vesicle in 5 min, *n* = 76 for S421S, 70 for S421A, and 77 for S421D. **j** Anterograde velocity, *n* = 72 for S421S, 70 for S421A, and 74 for S421D. Retrograde velocity, *n* = 76 for S421S, 70 for S421A, and 77 for S421D. Values were from three BRs and shown as mean ± SEM, and n.s., no significance.

A recent study suggested that phosphorylation at S421 may affect proteasome-dependent turnover of mHTT^24^. We examined HTT levels and found no significant differences in the expression levels of total HTT and mHTT protein across S421S, S421A, and S421D hiPSC lines (Fig. 3a, b), as well as NPCs (Fig. 3c–e) under normal culture conditions. After 24 h treatment with the proteasomal inhibitor Lactacystin (1 μM) or the lysosomal inhibitors ammonium chloride (NH_4_Cl, 20 nM) and Leupeptin (100 μM), we only observed increased mHTT in S421S NPCs treated with lysosomal inhibitors compared with the control group, but no changes in total HTT or wild-type (WT) HTT levels (Fig. 3c, f). This suggests the lysosomal pathway is an important pathway for mHTT degradation, and that S421A and S421D might increase lysosomal-dependent turnover of mHTT in our iPSC model. Furthermore, proteasomal and lysosomal inhibitors had no effect on total, WT, or mHTT levels in the S421A or S421D HD lines (Fig. 3c–e).

Several lines of evidence demonstrated that mHTT affects mitochondrial axonal transport in HD^25–27^. Here we tested whether mitochondrial trafficking was influenced by phosphorylation status at S421 using cortical neurons differentiated from S421S, S421A, and S421D HD hiPSCs (Fig. 3g). However, we did not find significant differences among S421S, S421A, and S421D neurons in the total axonal distance traveled in a 5 min interval (Fig. 3h), stationary events (Fig. 3i), and anterograde and retrograde velocity (Fig. 3j) of mitochondria.

Finally, we examined whether S421 phosphorylation status affects neural rosette formation. We have previously shown that the ability of hiPSCs to form neural rosettes is impaired by mHTT^14^. We performed the neural rosette formation assay on the S421S, S421A, and S421D hiPSC lines. We found that neither S421A nor S421D had an apparent effect on the deficits in neural rosette formation observed in HD hiPSCs (Supplementary Fig. S3).

### Analysis of differential gene expression in S421S and S421A/D NPCs

**Fig. 4:**
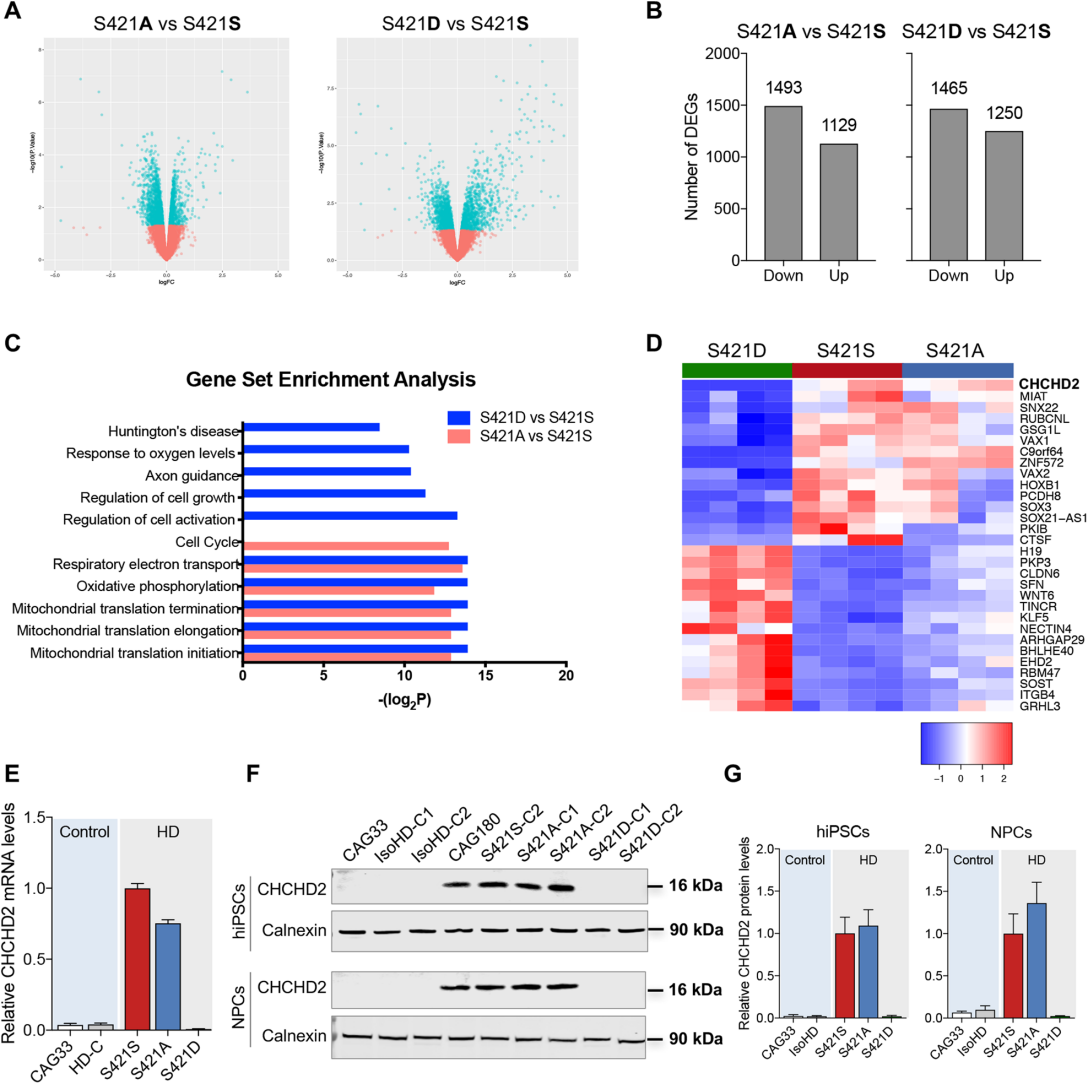
RNA-Seq analysis of genes differentially expressed in S421A and S421D relative to S421S HD neural cells. **a** Volcano plot of S421A vs. S421S and S421D vs. S421S (−log10(*p*-value) vs. log2FC), highlighting DEGs with nominal *p* < 0.05. **b** Number of up and down DEGs of S421A vs. S421S, and S421D vs. S421S with nominal *p* < 0.05. **c** GSEA. **d** Gene Venn analysis of specific DEGs of S421D vs. S421S with adjusted *p* < 0.05. **d** Heatmap of expression level for the top 30 genes only differentially expressed in S421D vs. S421S not in S421A vs. S421S. Heatmap represent four samples (two biological replicates from two independent clones) per genotype. **e** Expression levels of CHCHD2 in NPCs measured by qRT-PCR; Values shown as mean ± SEM; *n* = 3 BRs. **f** Expression levels of CHCHD2 in hiPSCs and NPCs measured by immunoblotting and relative quantification. **g** Values shown as mean ± SEM; *n* = 4 BRs for iPSCs and *n* = 3 BRs for NPCs (two subclones were used per genotype).

To gain insight into the cellular processes that might underlie the neuroprotection conferred by S421D relative to S421S and S421A, we performed RNA-Seq analysis on S421S, S421A, and S421D NPCs. For each genotype, two replicates from two individual clones were used for analysis of differentially expressed genes (DEGs). We identified a number of transcriptional gene expression changes associated with the S421 phosphorylation status (Fig. 4a). There were 1493 downregulated DEGs and 1129 upregulated genes in S421A vs. S421S NPCs, and 1465 downregulated genes and 1250 upregulated genes in S421D vs. S421S NPCs (Fig. 4b). GSEA results show that DEG-related mitochondrial translation, respiratory electron transport, and oxidative phosphorylation pathways were significantly enriched in both S421A and S421D gene expression signatures (Fig. 4c). The S421D gene expression signature was also significantly enriched for Huntington’s disease, response to oxygen levels, axon guidance, and regulation of cell activation/growth-related Gene Ontology (GO) terms (Fig. 4c). A clustered heatmap of the top 15 downregulated DEGs and top 15 upregulated DEGs is shown in Fig. 4d.

CHCHD2 is one of the top DEGs identified in our analysis of S421D vs. S421S NPCs and has been previously reported to be highly expressed in HD cells and tissues^14,28,29^. Therefore, we sought to further validate CHCHD2 expression in S421S and S421A/D hiPSCs and NPCs. Consistent with the RNA-Seq results, both mRNA levels of CHCHD2 (Fig. 4e) and CHCHD2 protein expression (Fig. 4f, g) were markedly higher in S421S and S421A cells compared with the isogenic control and S421D groups.

### S421 phosphorylation status affects mitochondrial morphology in NPCs

**Fig. 5:**
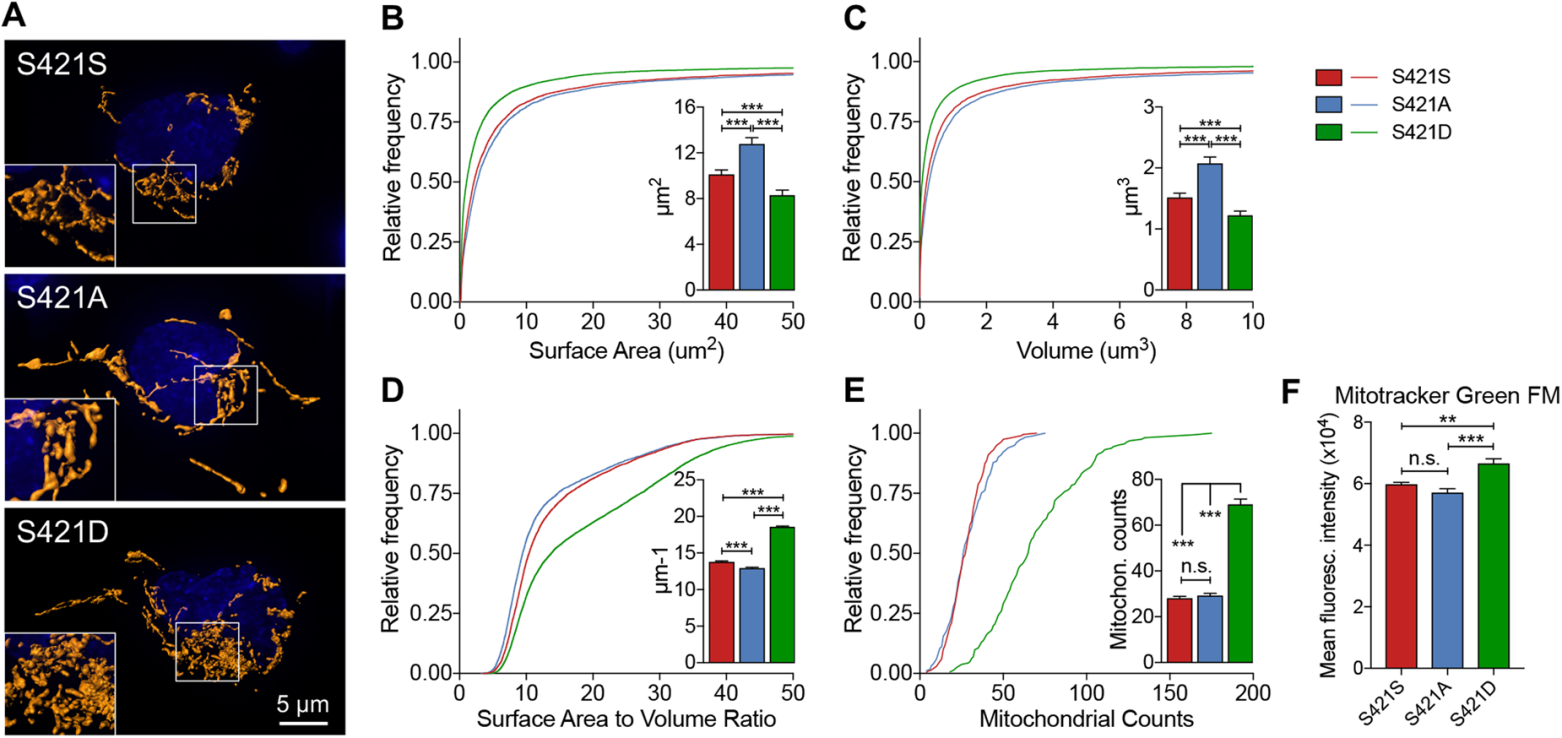
Phosphorylation status of S421-mHTT affects mitochondrial morphology and mass in HD hiPSC-derived NPCs. **a** Representative images of mitochondrial morphology in HD NPCs; Tom-20, orange; DNA, blue; scale bar = 5 μm. **b**–**e** Metrics of mitochondrial morphology in HD NPCs: surface area (**b**); volume (**c**); surface area to volume ratio (**d**); mitochondrial counts (**e**); *n* = 5599 mitochondria for S421S, 5463 mitochondria for S421A, 11,710 mitochondria for S421D from 2 different BRs (2 subclones were used per genotype). Values shown as mean ± SEM, n.s., no significance, and ***p < 0.001 was determined by Kruskal–Wallis one-way ANOVA with Dunn’s multiple comparisons test. **f** NPCs under basal culture conditions were stained with total mitochondrial marker Mitotracker Green FM; *n* = 6 for 2 clones per genotype with 3 BRs.

Animal and cell models of HD have provided compelling evidence that mitochondrial morphology and function are affected in HD^25,30^. Given that our transcriptional analysis suggested that mitochondrial pathways are influenced by S421 phosphorylation status, we sought to examine whether mitochondria are affected in S421D and S421A compared with S421S neural cells. We first imaged NPCs stained with TOM20, a mitochondrial marker, using high-resolution microscopy and then applied 3D reconstruction using the IMARIS software to quantify aspects of mitochondrial morphology. In support of the transcriptional analysis, we found a number of measures of mitochondrial morphology to be altered in S421D cells compared with S421S and S421A. S421D cells presented less mitochondrial surface area and volume, increased surface area to volume ratio, and more mitochondrial counts compared with S421S and S421A cells (Fig. 5a–e). Second, using established mitochondrial assays with fluorescent dyes in live cells^31,32^, we also observed increased total mitochondrial content in S421D NPCs (as measured by Mitotracker Green FM) when compared with S421S and S421A groups (Fig. 5f). These findings suggest that S421 phosphorylation status of mHTT affects processes that influence mitochondrial morphology.

### S421 phosphorylation status affects mitochondrial function in NPCs

**Fig. 6:**
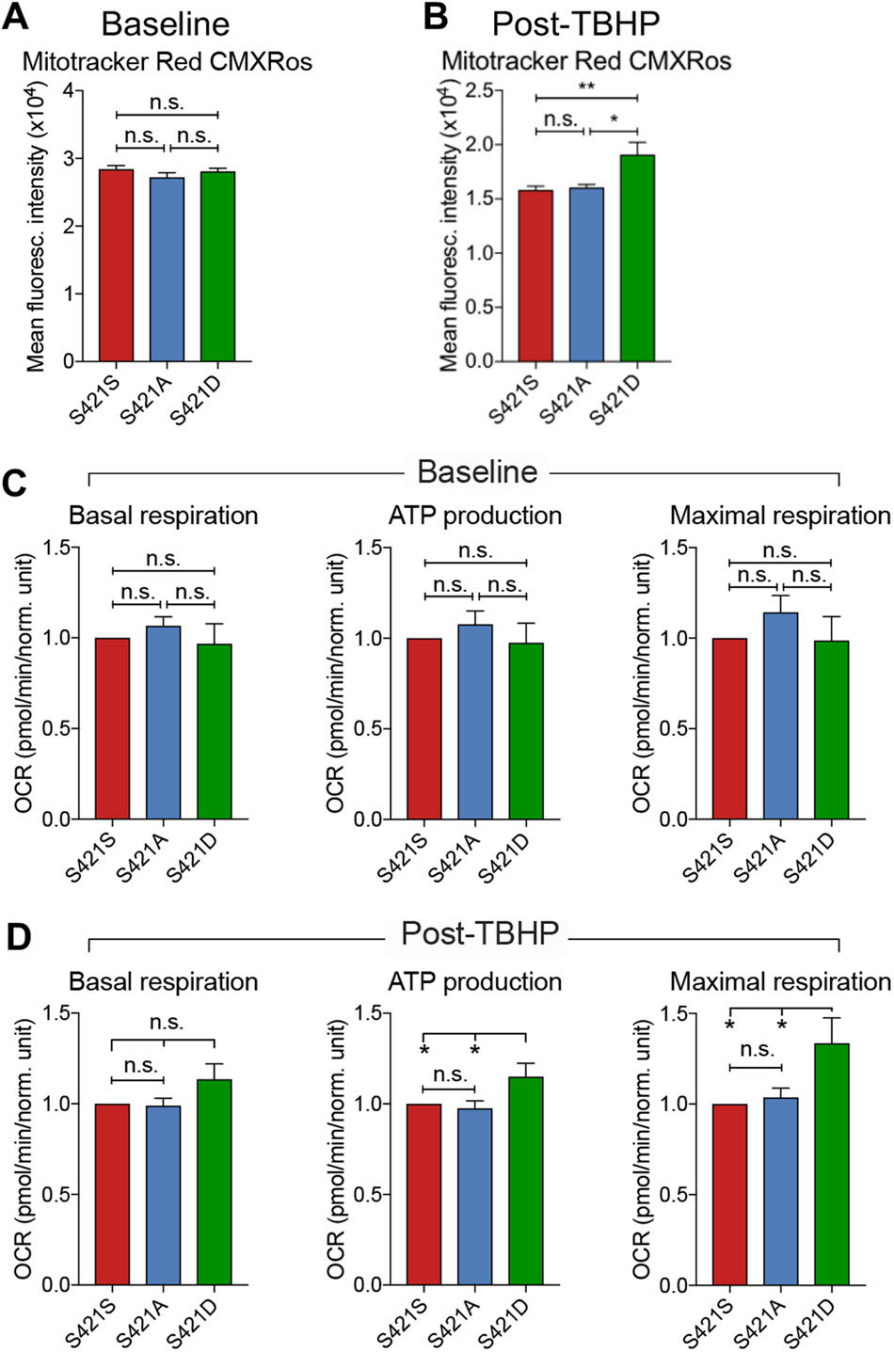
Mitochondrial function is affected by S421 phospho-status in HD neural cells. **a**, **b** NPCs were stained with functional mitochondrial marker Mitotracker Red CMXRos under basal (**a**) or oxidative stress (TBHP) (**b**) conditions; *n* = 6 for (**a**) and 8 for (**b**) for 2 clones per genotype with 3 BRs. Seahorse assay (adapted from Agilent website) used for measurements of mitochondrial respiration in NPCs under basal (**c**) and oxidative stress (TBHP) (**d**) conditions. Oxygen Consumption Rate (OCR) was calculated for basal respiration, ATP production, and maximal respiration; *n* = 3 for (C) and 5 for (D) BRs; Values shown as mean ± SEM and n.s., no significance, **p < 0.01 and ***p < 0.001 were determined by one-way ANOVA followed by a Fisher’s LSD post hoc multiple-comparison test.

To explore whether S421 phosphorylation status affects mitochondrial function, we first used Mitotracker Red CMXRos, a red fluorescent dye that stains mitochondria in live cells, to measure the amount of functional mitochondria. Although there was no difference between S421S, S421A, and S421D NPCs at baseline (Fig. 6a), the functional mitochondria content was higher in S421D NPCs when compared with S421S and S421A cells post-oxidative stress induced by TBHP treatment (Fig. 6b). Moreover, we further examined whether S421 status would affect mitochondrial respiration in HD NPCs, as we had previously observed decreased oxygen consumption rate in HD NPCs when compared with corrected isogenic controls^14^. Under basal culture conditions, we found no differences in mitochondrial basal respiration, ATP production, or maximal respiration rate in S421A or S421D NPCs compared with S421S NPCs (Fig. 6c). However, when exposed to oxidative stress, S421D cells displayed higher ATP production and maximal respiration rate compared with S421S and S421A NPCs (Fig. 6d). These findings are consistent with the Mitotracker Red CMXRos functional mitochondria results and indicate that the S421 phosphorylation status of mHTT not only affects mitochondrial morphology but also function.

## Discussion

We generated isogenic HD hiPSC lines that harbor phospho-resistant and pseudo-phosphorylated residues at the S421 site of mutant *HTT* using TALEN- and ssODN-mediated genome editing. These isogenic hiPSC lines contain full-length HTT alleles under an endogenous promoter in the context of human physiology, and thus represent relatively ideal models in which to study the role of S421 phosphorylation in HD. We found that S421D protected HD NPCs and neurons against cell death caused by TBHP treatment and GF withdrawal. Using genome-wide gene expression analysis, we observed that S421 phosphorylation status induced a number of gene expression changes, including ones associated with mitochondrial function. Consistent with results of the transcriptional analysis, we found that the phosphorylation status of S421 did indeed affect a number of mitochondrial properties including mitochondrial numbers and morphology. Although there were no differences between S421A and S421D at baseline, mitochondrial respiration was significantly improved in S421D but not S421A under oxidative stress. Here we provide an example of leveraging genome-editing technologies in hiPSCs to study the functional significance of PTMs in HD, which may be useful for the discovery and validation of potential PTM-targeted therapeutic approaches for HD.

Single PTMs can influence protein degradation and function, and thus impact disease phenotypes^2^. In this study, a single amino acid change (from serine to alanine or aspartic acid) at the S421-mHTT site resulted in robust effects on HD phenotypes. Importantly, S421A and S421D manifested different effects on disease phenotypes in different assays. First, S421D, but not S421A, demonstrated protective effects under TBHP oxidative stress or the GF withdrawal assay. In addition, S421D and S421A affected mitochondrial morphology in opposite directions. For example, S421D NPCs mitochondria showed smaller surface areas, while mitochondria in S421A NPCs had bigger surface areas than that of S421S NPCs. Second, S421A and S421D behaved in a similar way in assays of neural rosette formation and basal mitochondrial respiration where neither could rescue the deficits. Thirdly, RNA-Seq data showed both S421A and S421D could affect gene expression profile. Some DEGs were specific to either S421A or S421D, while a number of DEGs were common to both groups, which is consistent with their differential and similar effects in different pathways and phenotypes. One caveat to note when interpreting the differential effects of S421A vs. S421D is that the introduction of PTM-mimicking amino acid substitutions may impact the folding state and thus influence the toxicity of mHTT, a conformationally unstable protein, which may only partly mimic the effect of endogenous PTMs.

Several studies have demonstrated that S421D protected rodent striatal neurons against mHTT-induced toxicity in vitro and in vivo, whereas S421A aggravated the toxicity^9^. In addition, a recent study reported that S421D-mHTT transgenic mice had less severe motor and psychiatric-like behavioral deficits than S421S-mHTT (BACHD) mice from 3 to 12 months of age, while S421A-mHTT mice only showed motor deficits at a late stage (12 months old) compared to S421S-mHTT mice^24^. On the other hand, both S421A and S421D were reported to be protective in mouse primary cortical and striatal neuron-based assay of mHTT toxicity in earlier studies^33,34^. In our study, we observed that S421D protected hiPSC-derived NPCs and neurons against cell death induced by oxidative stress and GF deprivation. In contrast, susceptibility of S421A cells to cell death was similar to that of S421S cells. Furthermore, under normal neuronal culture conditions in the presence of GFs, S421A showed more cell death, while S421D had less cell death than S421S neurons. This discrepancy among studies likely reflects the different experimental paradigms employed, such as different cell types or animal models used in the studies, different HTT expression levels, and different HTT protein length.

Clearance of mHTT is primarily mediated by the ubiquitin-proteasomal system (UPS) and autophagy-lysosomal pathway^35^. The UPS predominantly degrades short-lived proteins by tagging these substrates with polyubiquitin chains, while autophagy is a cellular degradative pathway for long-lived cytoplasmic proteins, protein complexes/aggregates and damaged organelles^36^. However, it is still unclear which of the two systems is more efficient for mHTT clearance. A recent study using transgenic S421A vs. S421D HD mice suggested that phosphorylation mitigates neurodegeneration by increasing proteasome-dependent turnover of mHTT and reducing the presence of a toxic mHTT conformer^24^. However, we found no obvious changes in the steady-state levels of soluble full-length mHTT levels across S421S, S421A, and S421D cells generated by a seamless genome-editing approach. When evaluating the effects of S421 phosphorylation status on the turnover of mHTT, we observed greater accumulation of mHTT in S421S NPCs when inhibiting the lysosomal pathway. This suggests the lysosomal pathway is an important pathway for mHTT degradation, and S421A and S421D might increase lysosomal-dependent turnover of mHTT. This discrepancy with the previous study^24^ may be due to the different species, cellular systems, or nature of the mHTT protein (full length vs. fragment) employed.

Mitochondria are highly dynamic organelles that divide, fuse, and transport. These processes not only control mitochondrial morphology and number but also regulate mitochondrial function. Strong evidence indicates that mitochondrial morphology, transport and function are affected in HD^25^. However, there is no report on whether phosphorylation of HTT at S421 would affect mitochondrial morphology and function. In our study, we observed increased mitochondrial mass and smaller mitochondria in S421D NPCs, which may indicate increasing mitochondrial biogenesis and fission. Increased mitochondrial biogenesis is part of cellular response to oxidative stress, as mitochondrial biogenesis can attenuate oxidative stress by increasing mitochondrial capacity to metabolize reducing equivalents, which benefits cells with better survival^37,38^. Moreover, mitochondrial fission is required for removal of damaged and inactive organelles by autopay, which is important for maintenance of normal mitochondrial function^39^. Therefore, the mitochondrial mass and morphology changes in S421D cells might be an early compensative response to HD pathogenesis. In HD hiPSC-derived neural cells, we and others^24,40^ have observed deficits in mitochondrial respiration. In this study, we only observed rescuing effects of S421D on mitochondrial respiration of HD NPCs under oxidative stress but not basal conditions, suggesting that loss of phosphorylation of mHTT at S421 is more likely to be consequential under conditions of cellular stress.

CHCHD2, a member of “CHCHD” domain containing protein family, is involved in mitochondrial function, and plays important roles in the biogenesis and regulation of enzymes in the mitochondrial respiratory chain^41^. CHCHD2 regulates mitochondrial morphology in the fat cells of flies, and loss of CHCHD2 results in the increased mitochondrial fission^42^. In this study, we find the CHCHD2 expression is significantly decreased in S421D cells, which may account for the lower mitochondrial surface area and volume and higher mitochondrial counts in S421 NPCs, compared with the S421A and S421S groups.

It should be noted that electron microscopy analysis of mitochondrial ultrastructure would provide a more accurate assessment of mitochondrial morphological changes associated with S421-mHTT phosphorylation status than the super-resolution microscopy employed in the present study and should be considered in future follow-up studies.

In conclusion, our study demonstrates the significant impact of S421 phosphorylation status on mitochondrial form and function, as well as susceptibility to cell death in HD hiPSC-derived neural cells under stress conditions. Our results show that PTM at S421 may modulate the toxicity of the full-length mHTT protein at least in part by affecting HD-associated mitochondrial phenotypes. These findings provide further support that targeting phosphorylation of mHTT at S421 site can be considered for further development as a therapeutic strategy for HD.

## Supplementary information

Supplementary Figure 1

Supplementary Figure 2

Supplementary Figure 3

Supplementary Figure Legends

Supplementary Tables and Legends

## Data Availability

The datasets used and/or analyzed during the current study are included in this article and its Supplementary Information files. The datasets are available from the corresponding author on reasonable request.
