# Supplementary figures and images for: pS421 huntingtin modulates mitochondrial phenotypes and confers neuroprotection in an HD hiPSC model
Source: Cell Death Dis. 2020 Sep 25;11(9):809. doi: 10.1038/s41419-020-02983-z (PMC7519662; doi:10.1038/s41419-020-02983-z)

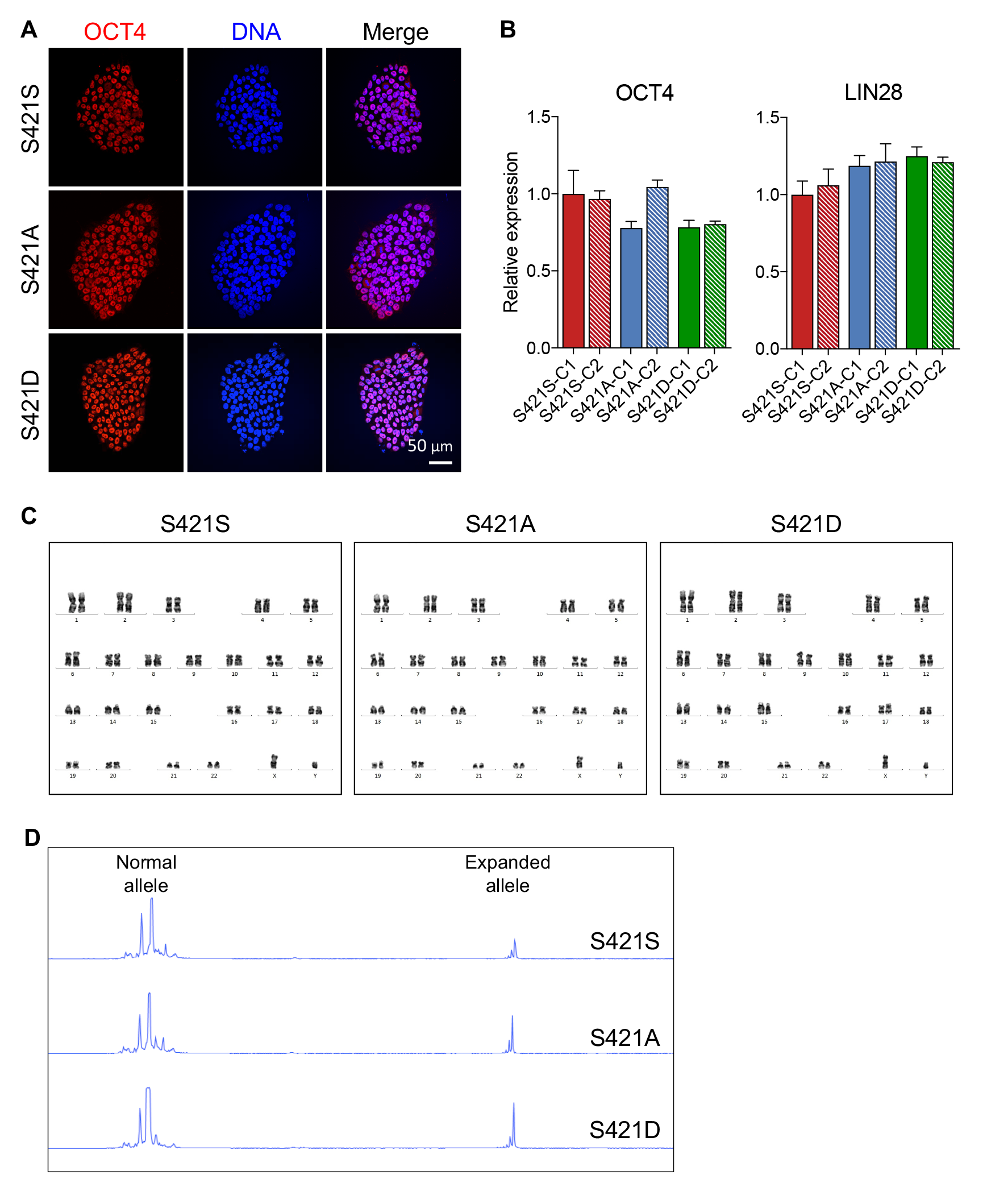

Supplement: Supplementary file 1 — Supplementary Figure 1 [file 41419_2020_2983_MOESM1_ESM.tif]

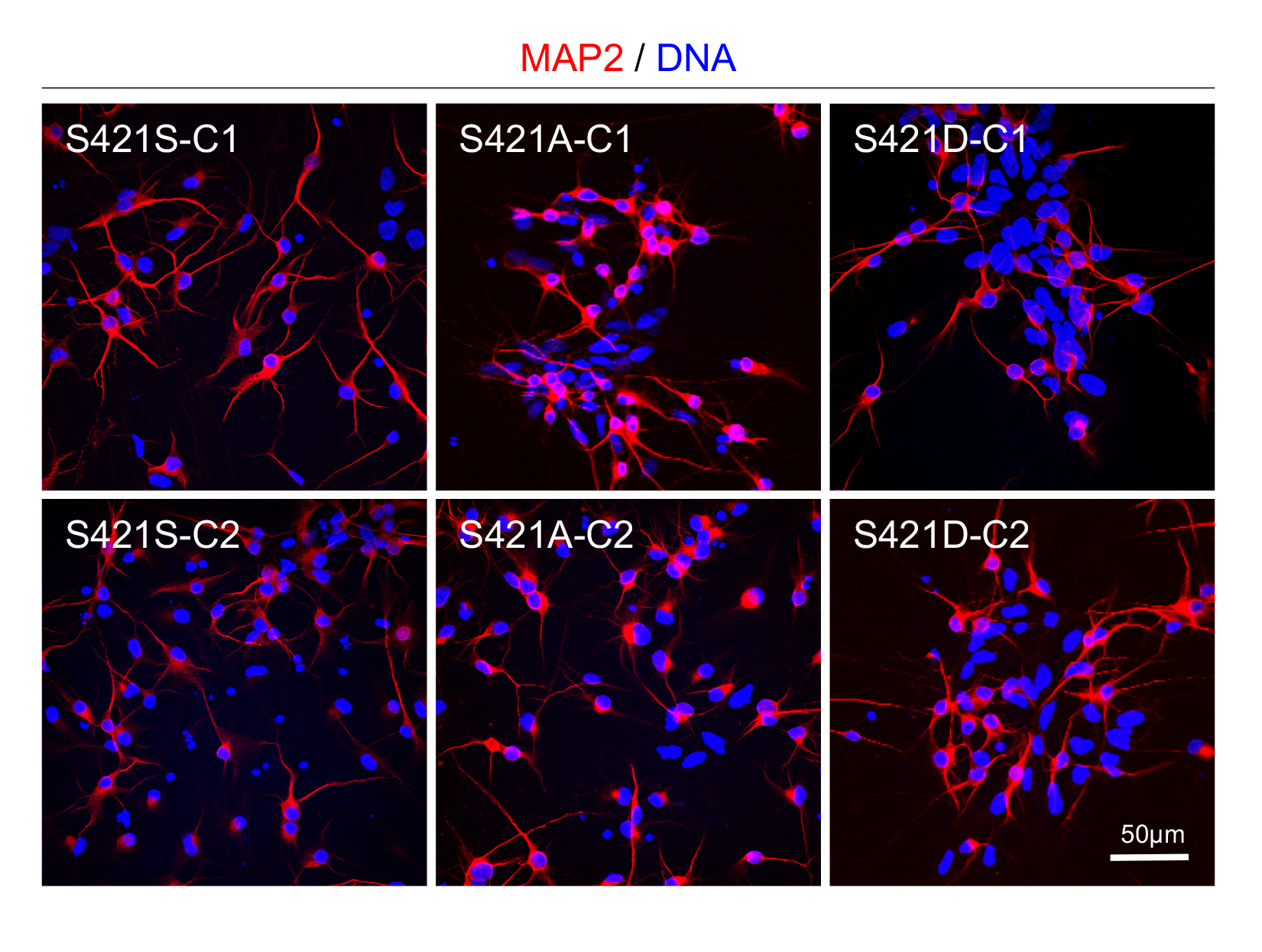

Supplement: Supplementary file 2 — Supplementary Figure 2 [file 41419_2020_2983_MOESM2_ESM.tif]

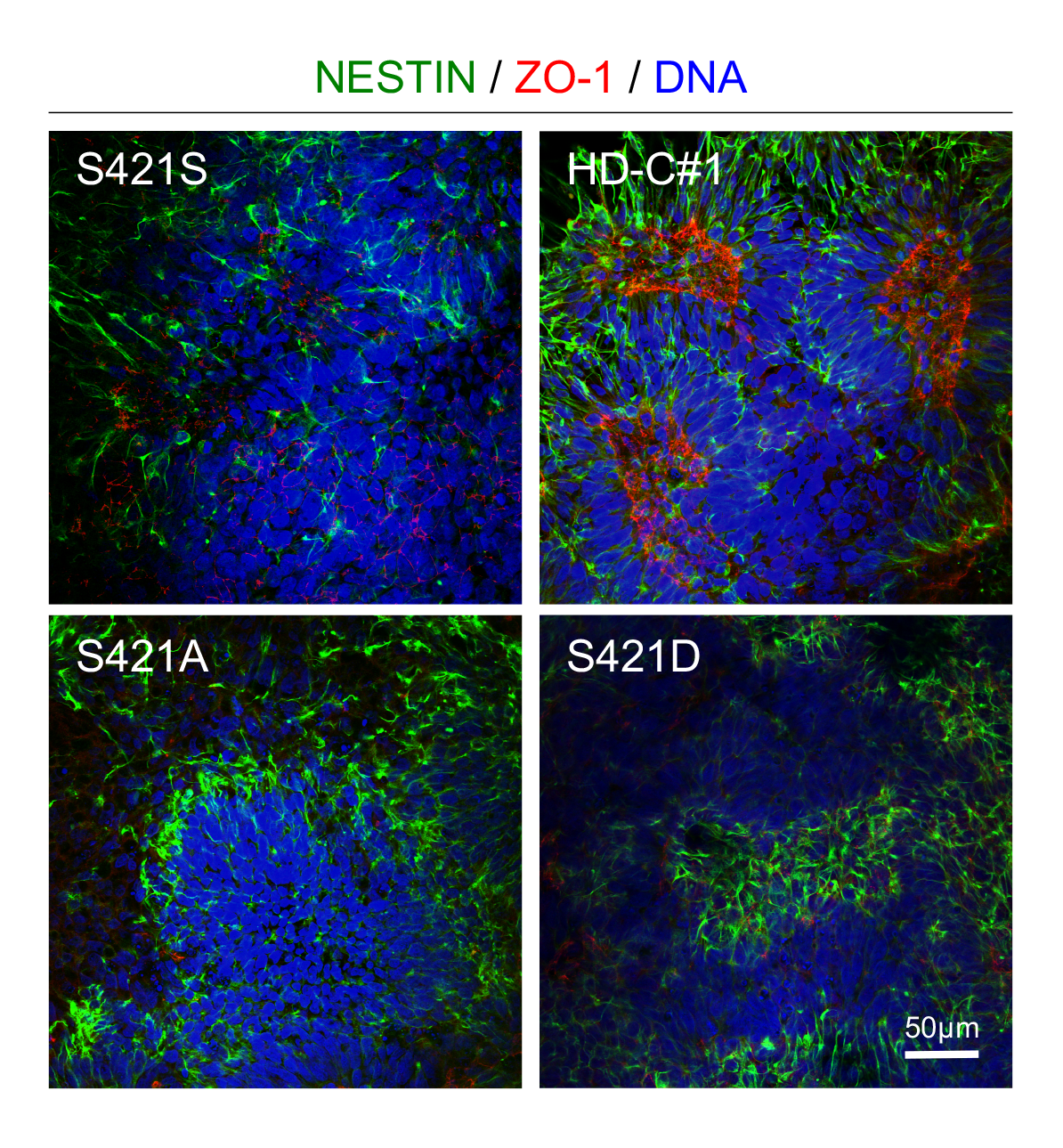

Supplement: Supplementary file 3 — Supplementary Figure 3 [file 41419_2020_2983_MOESM3_ESM.tif]
