# Supplementary material for: pS421 huntingtin modulates mitochondrial phenotypes and confers neuroprotection in an HD hiPSC model
Source: Cell Death Dis. 2020 Sep 25;11(9):809. doi: 10.1038/s41419-020-02983-z (PMC7519662; doi:10.1038/s41419-020-02983-z)
Supplement: Supplementary file 4 — Supplementary Figure Legends [file 41419_2020_2983_MOESM4_ESM.docx]

**Supplementary Figure Legends**

**Supplementary Fig. S1 Genome-edited HD hiPSC clones maintain pluripotency and normal karyotype.**

(**A-B**) The targeted HD hiPSCs maintain pluripotency as shown by positive immunostaining for the pluripotency marker OCT4 (**A**), and mRNA expression of pluripotency genes OCT4 and LIN28 (n= 3 per clone; values for independent biological replicates (BRs) shown as mean ± SEM) (**B**); (**C**) Karyotyping and g-band analysis show all targeted hiPSC clones have a normal (46, XY) karyotype; (**D**) CAG fragment sizing analysis plotted using Geneious 2 software.

**Supplementary Fig. S2 Confirmation of identity of hiPSC-derived neurons.**

hiPSC-derived neurons on Day 40 were stained with post-mitotic neuronal marker MAP2 (red) and DNA marker DAPI (blue); scale bar, 50 μm.

**Supplementary Fig. S3 No obvious effect of S421 status on mutant HTT-induced deficits in neural rosette formation.**

Neural rosettes were stained with neural stem cell marker NESTIN (green), luminal neural rosette marker ZO-1 (red), and nuclear DNA (blue); scale bar, 50 μm.
