# Supplementary material for: pS421 huntingtin modulates mitochondrial phenotypes and confers neuroprotection in an HD hiPSC model
Source: Cell Death Dis. 2020 Sep 25;11(9):809. doi: 10.1038/s41419-020-02983-z (PMC7519662; doi:10.1038/s41419-020-02983-z)
Supplement: Supplementary file 5 — Supplementary Tables and Legends [file 41419_2020_2983_MOESM5_ESM.docx]

**Supplementary Information**

**Materials and Methods**

**Nucleofection of hiPSCs**

hiPSCs were pre-treated with 10 µM Y-27632 for 2 h, and then were dissociated with Accutase, and 1 X 10^6^ cells were electroporated using the Neon Transfection System (Life Technologies) with 4 µg S421-T1, 4 µg S421-T2, and 6 µL S421A/D ssONDs (100µM) donor at 1,400 V for 2 pulses of 10 ms. Single GFP(+)/RFP(+) cells were sorted into 96-well plate coated with Matrigel by MoFlo™ XDP flow machine (Beckman Coulter) after 72 h post transfection. Surviving clones were expanded for culture and PCR screening.

**Screening targeted clones**

gDNA from the targeted clones were extracted using a published column-free method (Ramirez-Solis R, 1992). S421-F (5’-CAATGTTGTGACCGGAGCCCTG-3’) and S421-R (5’-ACAGGGTCTCACTTGGCCAGGTT-3’) primers were used to amplify the 420bp fragment flanking S421 site from gDNA. PCR products were digested using BglI restriction enzyme at 37°C for 2 h before being separated by agarose gel.

**Allele-specific PCR**

Allele-specific forward primers were designed for WT (F^WT^, 5’-TGGTGCCAGAAAGGTTGGGG-3’) and mutant allele (F^Mu^, 5’-TGGTGCCAGAAAGGTTGGGT-3’). PCR products by F^WT^/F^Mu^ and S421-R were purified for Sanger sequencing.

**Fragment sizing analysis**

To amplify the CAG tract, 100ng of cDNA of parental CAG180 and the isogenic S421S, S421A, and S421D hiPSC lines was used as template DNA with KOD Xtreme (Novagen, #71975) supplemented with 8% DMSO (final v/v). Cycling conditions were as follows: initial denaturation at 96°C for 5 mins, followed by 7 cycles of 96°C for 45s, 70°C for 30s, 72°C for 2 mins; and 33 cycles of 96°C for 45s, 58°C for 30s and 72°C for 2mins and a final elongation at 72°C for 10mins. Primers used for amplification span exons 1-6 of HTT cDNA and are 6-FAM conjugated L33FAM-5’-CGAGTCCCTCAAGTCCTTCC-3’ and unconjugated R390-5’-TTCCATAGCGATGCCCAGAA-3’. Amplicons were visualized on 1% agarose gel on the Geldoc XR system (Bio-Rad) and sent to Laragen Inc (CA, US) for fragment sizing with the GeneScan™ 1200 LIZ® dye Size Standard.

**RNA isolation, cDNA synthesis, and qPCR**

Cells were lysed using RLT Plus Buffer and RNA was purified using the RNeasy Plus Mini kit (QIAGEN) according to the manufacturer’s instructions. For all samples, cDNA was generated using PrimeScript® RT reagent Kit (TAKARA). Quantitative real-time PCR was performed on the StepOnePlus™ or QuantStudio 6 Flex Real-Time PCR System (Applied Biosystems) using primers listed in **Table S3**. Relative gene expression levels were analyzed using the comparative CT Method (ΔΔCT Method).

**CellTiter-Glo® luminescent cell viability assay**

NPCs were seeded on the 96-well plate at a density of 1 X 10^5^ cells per well one day before TBHP treatment. Next day, NPCs were incubated with 200 µM TBHP for 2 h and cultured for another 24 h, and then cells were assayed using CellTiter-Glo***®*** Luminescent Cell Viability kit (Promega, G7571) according to manufacturer’s instructions.

**Growth factor withdrawal assay**

The forebrain neuronal cells on Day 40 were switched to N2B27 medium supplemented 50 ng/mL BDNF or basal medium only for 48 h. Cells were then fixed with 4% paraformaldehyde (PFA) and stained with TUNEL (Roche, In Situ Cell Death Detection Kit). TUNEL (+) and DAPI (+) cells were counted using Image J software and the data from two individual clones per genotype were combined together for statistical analysis.

**Measurement of mitochondrial morphology**

Samples for mitochondrial morphology measurement were prepared the same way as immunofluorescence staining samples, except that 18 mm diameter #1.5H glass coverslips were used. 2.5 x 10^5^ NPCs were seeded per well of 12-well plate and coverslips mounted on glass slides with H-1000 Vectashield anti-fade mounting medium (Vector Laboratories, USA). Cells were fixed and immunostained with anti-rabbit TOM20 (1:500; Proteintech, 11802-1-AP) primary antibodies and Alexa Fluor 568-conjugated animal anti-rabbit IgG (1:1000) secondary antibodies to visualize the mitochondrial network.

A DeltaVision OMX v4 Blaze microscope (GE Healthcare, Issaquah, WA, USA) equipped with a solid-state illuminator for widefield excitation and the BGR-FR filter drawer was used for acquisition of widefield-deconvolved images of individual NPCs. An Olympus Plan Apochromat 100x/1.4 Point Spread Function (PSF) oil immersion objective lens was used with liquid-cooled Evolve EM-CCD cameras (Photometrics, Tucson, AZ, USA) for each channel. Images were acquired at a z-spacing of 0.125 µm. Deconvolution of the widefield images, followed by chromatic alignment, was conducted using the SoftWorX (GE Healthcare) program*.*

For each genotype, a total of 40 to 50 images per slide were captured and this was repeated with biological replicates. Excitation intensity was kept constant for all channels and all samples. To measure mitochondrial morphological parameters, the deconvolved widefield images were imported into IMARIS (Bitplane) for 3D reconstruction. Masking was done to define the boundary of each cell to ensure that each image contained only one cell for ease of quantification. All images were then processed with the following parameters: no smoothing of surface, automatic filtering and automatic signal detection by k-mean values of images. Raw data points of each mitochondrion detected were exported for statistical analysis.

**RNA-Seq analysis**

RNA was extracted from cells using a RNeasy plus mini kit (Qiagen) according to the manufacturer’s instructions. Subsequent library preparation and paired-end 100bp sequencing and 30M reads/per sample using Illumina HiSeq4000-PE150 were performed by Novogene (Hong Kong). To align the FASTQ files, Star v.2.5.0a was used against the GRCm38 primary assembly annotation. In order to identify outliers, PCA plots of the samples were used. A cutoff of 10 reads on average per gene was used a filter. CalcNormFactors from the edgeR v.3.18.1 R package was used to normalize the counts. The limma v3.32.10 R package was used to transform and model the gene-level differential expression data. limma::voom was used to transform the normalized count data to log2-counts per million (CPM) and calculate the mean-variance relationship. limma::lmFit was used to fit a linear model for each gene based on the experimental design matrix. limma::eBayes was used to calculate the empirical Bayes moderated t-statistic for contrast significance. Multiple hypothesis adjusted p-values were calculated using limma::toptable, which implemented the Benjamini-Hochberg procedure to control FDR. To create heatmaps, heatmap3 v.1.1.1 R package was used, and to build volcano plots, ggplot2 v2.2.1 R package was used. Gene Set Expression Analysis (GSEA) was used to test whether the gene expression signatures of pS421-mHTT were enriched for KEGG, Reactome, and Gene Ontology Biological Process pathways.

**Supplementary Tables and Legends**

**Supplementary Table S1. Sequences of S421 TALEN binding sites and ssODNs**

| Name | 5’-3’ sequence |
| --- | --- |
| S421-T1 | CTGGTGGCCGAAGCCGT |
| S421-T2 | TGGAACTTATAGGCAAGTT |
| S421A-ssODNs | AGCTCACCGCTGCTAAGGAGGAGTCTGGTGGCCGAAGCCGTAGTGGCGCTATTGTGGAACTTATAGGCAAGTTATTAGCAAGGTCTACTC |
| S421D-ssODNs | AGCTCACCGCTGCTAAGGAGGAGTCTGGTGGCCGAAGCCGTAGTGGCGATATTGTGGAACTTATAGGCAAGTTATTAGCAAGGTCTACTC |

**Supplementary Table S2. Off-target analysis for *HTT* S421 TALENs**

| No. | Chr. | Start | Stop | Spacer | S421-T1 sequence | S421-T2 sequence | In exons |
| --- | --- | --- | --- | --- | --- | --- | --- |
| 1 | 5 | 162843419 | 162843465 | 13 | TTATGTGAAAGAAGCACT | TAAATTGCCCATAAGCTT | No |
| 2 | 6 | 105402172 | 105402218 | 13 | TTCTGTGAAAGAAGCACT | TAAATTGCCCATAAGCTT | No |
| 3 | 6 | 157730905 | 157730955 | 17 | TAAATTATCTGTAAATTC | TCTAGAGAATGCATTCAT | No |
| 4 | 10 | 56905944 | 56905989 | 12 | TAACAGCCCTATAACTTA | TCATTTGGCGAAACAAGT | No |
| 5 | 9 | 80349158 | 80349208 | 17 | TATCTTGCCAATAAGAAC | TTTGCTAAGAAAAGAAGT | No |

**Supplementary Table S3. Sequences of primers for qRT-PCR analysis**

| Name | Forward (5ʹ→3ʹ) | Reverse (5ʹ→3ʹ) |
| --- | --- | --- |
| *ACTIN* | GGCATGGGTCAGAAGGATTC | CACACGCAGCTCATTGTAGAAG |
| *OCT4* | AGTTTGTGCCAGGGTTTTTG | ACTTCACCTTCCCTCCAACC |
| LIN28 | GCGGGCATCTGTAAGTGGTT | GGTGAACTCCACTGCCTCAC |
| *CHCHD2* | GCTTCAGTGGAGGAAGTAATG | TGATGTCACCCTGGTTCT |
